# Analysis of functional xylanases in xylan degradation by *Aspergillus niger* E-1 and characterization of the GH family 10 xylanase XynVII

**DOI:** 10.1186/2193-1801-2-447

**Published:** 2013-09-09

**Authors:** Yui Takahashi, Hiroaki Kawabata, Shuichiro Murakami

**Affiliations:** Department of Agricultural Chemistry, Graduate School of Agriculture, Meiji University, 1-1-1, Higashimita, Tama-ku, Kawasaki, 214-8571 Japan; Department of Agricultural Chemistry, Faculty of Agriculture, Meiji University, 1-1-1, Higashimita, Tama-ku, Kawasaki, 214-8571 Japan

**Keywords:** *Aspergillus niger*, Endo-β-1,4-xylanase, Glycoside hydrolase family 10, Xylan degradation

## Abstract

Xylanases produced by *Aspergillus niger* are industrially important and many types of xylanases have been reported. Individual xylanases have been well studied for their enzymatic properties, gene cloning, and heterologous expression. However, less attention has been paid to the relationship between xylanase genes carried on the *A. niger* genome and xylanases produced by *A. niger* strains. Therefore, we examined xylanase genes encoded on the genome of *A. niger* E-1 and xylanases produced in culture. Seven putative xylanase genes, *xynI*–*VII* (named in ascending order of the molecular masses of the deduced amino acid sequences), were amplified from the strain E-1 genome using primers designed from the genome sequence of *A. niger* CBS 513.88 by PCR and phylogenetically classified into three clusters. Additionally, culture supernatant analysis by DE52 anion–exchange column chromatography revealed that this strain produced three xylanases, XynII, XynIII, and XynVII, which were identified by N-terminal amino acid sequencing and MALDI-TOF-MS analyses, in culture when gown in 0.5% xylan medium supplemented with 50 mM succinate. Furthermore, XynVII, the only GH family 10 xylanase in *A. niger* E-1, was purified and characterized. The purified enzyme showed a single band with a molecular mass of 35 kDa by SDS-PAGE. The highest activity of purified XynVII was observed at 55°C and pH 5.5. The enzyme was stable in the broad pH range of 3–10 and up to 60°C and was resistant to most metal ions and modifying regents. XynVII showed high specificity against beechwood xylan with *K*_m_ and *V*_max_ values of 2.8 mg mL^–1^ and 127 μmol min^–1^mg^–1^, respectively. TLC and MALDI-TOF-MS analyses showed that the final hydrolyzed products of the enzyme from beechwood xylan were xylose, xylobiose, and xylotriose substituted with a 4-*o*-metylglucuronic acid residue.

## Introduction

Xylanolytic enzymes have great biotechnological potential for various industrial processes, including feed, food, pulp, and others (Akpinar et al. 2009; Beg et al. 2000; Camacho and Aguilar 2003; Twomey et al. 2003), and there has been much interest in microorganisms that produce the enzymes, properties of the produced enzymes, and their genes, from applicable standpoints. Many studies on the utilization of the microorganisms, their enzymes, and reaction products make progress in the broad range of industrial applications, and commercial xylanases have been industrially produced in many countries (Beg et al. 2001).

Xylan is the most abundant hemicellulose and a complex polysaccharide composed of a backbone of β-1,4-glycoside–linked xylose residues. Several hydrolytic enzymes are required for complete xylan degradation. Endo-1,4-β-xylanase (EC. 3.2.1.8) plays a major role in the degradation of xylan by cleaving the xylosyl backbone and releasing short xylooligosaccharides, which are further hydrolyzed into xylose units by xylan 1,4-β-xylosidase (EC. 3.2.1.37) (Shallom and Shoham 2003). Many microorganisms, including bacteria, fungi, and yeast, are known to produce these xylanolytic enzymes (Beg et al. 2001). The filamentous fungi *Aspergillus* and *Penicillium* are particularly important xylanase producers because they excrete the enzyme into media at higher levels than other microorganisms (Chávez et al. 2006; de Vries and Visser 2001). Since the xylanases from *Aspergillus niger*, an excellent xylanase producer, were first purified and characterized in 1977 (Gorbacheva and Rodionova 1977), many xylanase purification has been reported, and they were found to differ in molecular masses and pIs (Fournier et al. 1985; Frederick et al. 1981; Frederick et al. 1985; Shei et al. 1985). To date, many xylanase genes have been cloned from newly isolated *A. niger*, strain and enzymes expressed from the cloned genes were characterized in detail (Deng et al. 2006; Kinoshita et al. 1995). Detergent and organic solvent-stable xylanase has also been studied toward industrial applications (Hmida-Sayari et al. 2012).

In the 21^st^ century, genome sequencing of microorganisms has steadily revealed exhaustive information about the genes found on microbial genomes. The genome sequence of *A. niger* reported by Pel et al. (2007) includes five candidate xylanase genes: one xylanase belonging to the glycoside hydrolase (GH) family 10 and four other genes homologous to GH family 11 enzymes. Although genome-wide knowledge and enormous individual data on various xylanases and their genes from *A. niger* have been generated, it is unclear how many xylanase genes are encoded on each genome of isolated *A. niger* strains and which xylanases are produced in culture. Thus, to our knowledge, there are very few studies that have exhaustively linked the putative xylanase genes encoded on *A. niger* genome to all xylanases secreted into culture. Furthermore, it appears that the physiological roles of each xylanase in xylan degradation remain poorly characterized. We, therefore, aimed to clarify the relationship between xylanase genes encoded on the genome and xylanases produced in culture.

In a previous study, we isolated *A. niger* E-1, which produces a high level of xylanase, from gray gentle lemur feces extracts (Takahashi et al. 2012). This report describes the characterization of xylanase genes on the *A. niger* E-1 genome. In addition, we found three xylanases (XynII, XynIII, and XynVII) in the culture supernatant of *A. niger* E-1 and purified and characterized XynVII because little was known about this enzyme.

## Materials and methods

### Microorganisms and culture conditions

*Aspergillus niger* E-1 was previously isolated from gray gentle lemur feces extracts (Takahashi et al. 2012). For xylanase production, a shaking flask (500 mL) containing 50 mL of 0.5% beechwood xylan (Sigma-Aldrich, St. Louis, MO, USA) medium (pH 5.5) (Takahashi et al. 2012) supplemented with 50 mM sodium succinate was inoculated with spores (5 × 10^6^). Inoculated flasks were incubated at 130 rpm and 37°C. After filtration and centrifugation, the culture supernatant was used as a crude enzyme preparation.

*Escherichia coli* XL1-Blue was used in molecular biological experiments and cultured in Luria–Bertani medium (Sambrook and Russell 2001) supplemented with ampicillin (100 μg mL^–1^) and tetracycline (12.5 μg mL^–1^), and when necessary, isopropyl β-D-thiogalactopyranoside (1 mM) and X-Gal (0.04%) at 37°C under aerobic conditions.

### Cloning of xylanase genes from *A. niger* E-1

To design primers to amplify putative xylanase genes and their homologues encoded on *A. niger* E-1 genome, xylanase genes on *A. niger* CBS 513.88 genome that was sequenced completely (Pel et al. 2007), were searched at databases of DNA Data Bank of Japan (DDBJ) using an All-round Retrieval of Sequence and Annotation Program. We found eight candidate xylanase genes, including one gene for GH family 10 xylanase, four genes for GH family 11, and three putative genes showing weak similarity to endoxylanases of other microorganisms. Information on these putative enzymes is summarized in Table 1. We designed primer pairs on the basis of the 5′- and 3′-terminal sequences of the candidate xylanase genes (Table 2). To amplify *xynVIII*, another primer pair, xynVIII2F and xynVIII2R was also designed on the basis of the internal sequence of putative *xynVIII*.

**Table 1:** **Putative xylanases encoded on*A. niger*CBS 513.88 genome**

Name	Deduced MM (kDa)^a^	pI^a^	Accession no.	Protein ID
XynI	19.0	3.97	AM270363-19	CAK42832.1
XynII	22.6	4.10	AM270327-78	CAK46731.1
XynIII	24.1	5.09	AM269952-5	CAK43456.1
XynIV	24.9	3.77	AM269993-14	CAK44157.1
XynV	27.9	4.56	AM270343-22	CAK97322.1
XynVI	35.4	4.45	AM270229-8	CAK40644.1
XynVII	35.5	6.19	AM270045-11	CAK38067.1
XynVIII	86.1	4.40	AM270362-17	CAK48600.1

^a^Each value was calculated on the basis of amino acid sequence containing a putative signal peptide sequence.

**Table 2:** **Primers designed from putative xylanase gene sequences of*A. niger*CBS 513.88**

Gene	Size (bp)^a^	Primer name	Primer sequence
*xynI*	543	xynIF	5′-atgttcttcaagaccatcctt
		xynIR	5′-ttaacccagagtaagagtgaa
*xynII*	686	xynIIF	5′-atgaaggtcactgcagcttt
		xynIIR	5′-taagaagatatcgtgacactg
*xynIII*	746	xynIIIF	5′-atgctcaccaagaaccttct
		xynIIIR	5′-ttactgaacagtgatggacg
*xynIV*	755	xynIVF	5′-tggtcgcctactcgtctc
		xynIVR	5′-tagcagctctcctcggtg
*xynV*	826	xynVF	5′-atggtgtctttccttggcc
		xynVR	5′-ctatgcactaacggtgaagt
*xynVI*	1043	xynVIF	5′-atgtcgcacccccaacag
		xynVIR	5′-ctacgataaagtcctcccc
*xynVII*	1494	xynVIIF	5′-atggttcagatcaaggtagc
		xynVIIR	5′-ctagagagcatttgcgatag
*xynVIII*	2962	xynVIIIF	5′-tgcgcgtaccgaaccgg
		xynVIIIR	5′-tcatatgctccgatgtccc
		xynVIII2F^b^	5′-aacggaggcatacctcaac
		xynVIII2R^b^	5′-tgttgccacccagcagac

^a^Putative intron sequences are included in gene sizes.

^b^xynVIII2F and xynVIII2R were designed from the internal sequence of *xynVIII.*

Mycelia of *A. niger* E-1 were harvested from culture grown for 72 h by filtration, and total DNA was extracted by the method of Hamamoto (2001), except for the extraction buffer. We used an extraction buffer of 40 mM ethylene-*N*,*N*,*N′*,*N′*-diaminetetraacetic acid disodium salt dihydrate (EDTA) and 100 mM Tris–HCl (pH 9.0). Xylanase genes of strain E-1 were amplified by polymerase chain reaction (PCR) using appropriate primer pairs shown in Table 2. PCR was performed using TaKaRa Ex Taq (Takara Bio, Ohtu, Japan). The temperature profile consisted of an initial denaturation step of 2 min at 98°C, followed by 30 cycles of denaturing at 98°C for 30 sec, annealing at 53°C for 30 sec, and extension at 72°C for 4 min. The amplified products were cloned and sequenced by the methods described previously (Takahashi et al. 2012). The sequences obtained were compared with those of the putative *A. niger* CBS 513.88 xylanase genes using GENETYX-WIN version 3.1.0 (Software Development, Tokyo, Japan).

Multiple sequence alignment and phylogenetic tree construction were performed using ClustalW 2.1 software at the DDBJ. The phylogenetic tree was visualized using TreeView 1.6.6 software available online (http://taxonomy.zoology.gla.ac.uk/rod/treeview.html). The DDBJ/EMBL/GenBank accession numbers for the reported sequences in this paper are AB821364–AB821370.

### Enzyme assays

Xylanase activity was assayed as described previously (Takahashi et al. 2012). One unit (U) of xylanase activity was defined as the amount of enzyme that releases 1 μmol of reducing sugar as a xylose equivalent per minute. Protein concentrations were estimated by the method of Lowry et al. (1951) using bovine serum albumin (Wako Pure Chemical Industries, Osaka, Japan) as a standard.

### Separation and purification of xylanases

The crude enzyme preparation (4,500 U) was concentrated by lyophilization and dissolved in 20 mM acetate buffer (pH 5.5; buffer A). The concentrated sample was dialyzed against buffer A and applied to a DE52 (Whatman, Kent, UK) anion-exchange column (1.4 × 13 cm) equilibrated with buffer A at a flow rate of 50 mL h^–1^. Unbound proteins were eluted with buffer A and collected as an active xylanase fraction (fraction 1). Purity of the fraction was examined by native- and sodium dodecyl sulfate (SDS)-polyacrylamide gel electrophoresis (PAGE), and this fraction was used for further characterization experiments. Bound proteins were eluted with a liner gradient of NaCl (0–0.5 M) at a flow rate of 50 mL h^–1^, and the eluate was separated into tubes (3.2 mL for each). Enzyme activities and protein concentrations were examined in fraction 1 and collected tubes.

### Native-PAGE and zymogram analysis

Native-PAGE was performed by the method of Davis (1964) using a 7.5% (w/v) acrylamide gel with Tris–glycine buffer (pH 8.3). After running, the gel was stained with Coomassie brilliant blue R-250 (CBB) or, for zymogram analysis, soaked in 83 mM acetate buffer (pH 5.5) containing 0.4% xylan for 30 min at room temperature with gentle shaking and then incubated for 1 h at 37°C. After incubation in 0.1% (w/v) Congo Red for 15 min at room temperature, the gel was washed with 1 M NaCl and then soaked in 5% acetic acid to visualize clear bands with xylanase activity.

### SDS-PAGE and pI determination

SDS-PAGE was performed by the method of Weber and Osborn (1969) using a 12% (w/v) acrylamide gel with Tris–glycine buffer (pH 8.3) containing 0.1% (w/v) SDS. Molecular weight marker, middle range (Wako Pure Chemical Industries) was used as a molecular mass standard. After electrophoresis, the gel was stained with CBB. Isoelectric focusing was performed using an Immobiline DryStrip with a pH range of 6–9 (18 cm; GE Healthcare, Buckinghamshire, UK) equipped with an Immobiline DryStrip kit (GE Healthcare). Electrophoresis was performed using a Multiphor II Electrophoresis System according to the manufacturer’s instructions (GE Healthcare). The isoelectric point was estimated from the position of a protein band on the Immobiline DryStrip after staining with CBB.

### N-terminal amino acid sequencing

After SDS-PAGE, proteins were electroblotted onto a Fluoro Trans membrane (Pall, Port Washington, NY, USA) using a Nihon Eido semidry type electroblotter NA-1512 (Nihon Eido, Tokyo, Japan) according to the manufacturer’s instructions. The membrane was stained with CBB, and protein bands were cut out. The protein bands were sequenced using a Shimadzu protein sequencer PPSQ-31A (Shimadzu, Kyoto, Japan).

### Protein identification by MALDI-TOF-MS

To identify purified protein, a gel slice containing a CBB-stained protein band was excised after SDS-PAGE and subjected to digestion with sequencing grade modified trypsin (Promega, Madison, MI, USA) according to the method of Hong et al. (2011). The digested sample and standard peptides for analysis by Matrix-assisted laser desorption-ionization time-of-flight mass spectrometry (MALDI-TOF-MS) were prepared using a ProteoMass Peptide & Protein MALDI-MS Calibration Kit (Sigma-Aldrich) according to the manufacturer’s instructions as follows. One microliter of sample containing peptides, which were extracted from the gel slice after trypsin digestion, was mixed with 1 μL of matrix solution [10 mg mL^–1^ α-cyano-4-hydroxyciannamic acid in 0.1% trifluoroacetic acid solution–acetonitrile (1:1)] on a sample plate and then air-dried under dark condition. MALDI-TOF-MS analysis was performed by a Shimadzu MALDI-TOF-MS AXIMA Performance (Shimadzu) after calibration with bradykinin fragment 1–7 and insulin oxidized B chain (bovine) [(M + H)^+^: 757.3997 and 3,494.6513, respectively, as monoisotopic molecular masses] as peptide standards. For protein identification, MS spectra data obtained from MALDI-TOF-MS analysis were used to search for protein candidates in the SwissProt database using Mascot software programs (Matrix Science, Boston, MA, USA).

### Effects of temperature and pH on xylanase activity and stability

The effects of temperature on xylanase activity were examined under the standard assay conditions, expect that the reaction temperature ranged from 30°C to 70°C. The optimum pH was determined in 20 mM buffers adjusted to different pH, as described in the appropriate figure legend. In order to examine thermostability and pH stability, purified enzyme (1.5 U and 0.25 U, respectively) was incubated at various temperatures for 30 min or in 100 μL of 150 mM buffers adjusted to different pH, for 24 h at 4°C. The residual xylanase activities were assayed under standard conditions.

### Effects of metal ions and modifying reagents, and substrate specificity

The effects of chemicals on xylanase activity were examined in the presence of the various chemicals at the final concentration of 2 mM. Relative enzyme activity was calculated as the percentage of activity compared with that without any chemicals.

Cellulolytic and xylanolytic enzyme activities were assayed in a reaction mixture containing oatspelt xylan (Sigma-Aldrich), carboxymethylcellulose sodium salt (CMC, Sigma-Aldrich), crystalline cellulose (Sigmacell Cellulose, Type 20; Sigma-Aldrich), pectin from citrus (Wako Pure Chemical Industries), inulin (Tokyo Chemical Industry, Tokyo, Japan), dextran (Nacalai Tesque, Kyoto, Japan), or dextrin from corn (Sigma-Aldrich) instead of beechwood xylan. Enzyme activity was estimated by measuring the increase in reducing sugar released from each substrate. We also tested *p*-nitrophenyl α-L-arabinofuranoside (PNPAra, Sigma-Aldrich), *p*-nitrophenyl β-D-cellobioside (PNPC, Sigma-Aldrich), *p*-nitrophenyl β-D-glucopyranoside (PNPG, Nacalai Tesque), and *p*-nitrophenyl β-D-xylopyranoside (PNPX, Nacalai Tesque) using method described previously (Takahashi et al. 2012). Substrate specificity was expressed as relative percentages compared with beechwood xylan as a substrate.

### Kinetic parameters

Kinetic parameters were determined using several concentrations (1.25–10 mg mL^–1^) of beechwood xylan in 83 mM acetate buffer (pH 5.5) under standard assay conditions. The Michaelis–Menten constant (*K*_m_) and maximal reaction velocity (*V*_max_) values were estimated by linear regression from double-reciprocal plots according to the method of Lineweaver and Burk (Lineweaver and Burk 1934).

### Analysis of hydrolyzed products

The hydrolyzed products from beechwood xylan were examined by thin-layer chromatography (TLC). Purified xylanase (10 U mL^–1^) was incubated in 20 mM buffer A containing 1.7% beechwood xylan, xylobiose (X2), xylotriose (X3), xylotetraose (X4), xylopentaose (X5), or xylohexaose (X6) at 37°C for an appropriate time and the enzyme reactions were stopped by boiling. After centrifugation, the supernatants were concentrated by a vacuum centrifuge and spotted onto a silica gel 60 F254 aluminium plate (Merck, Whitehouse Station, NJ, USA), developed using the solvent system of acetonitrile–ethyl acetate–2-propanol–water (17:5:11:10, v/v/v/v). Xylose and X2–X6 were used as standards. The hydrolysates were detected by spraying with 10% (v/v) sulfuric acid in methanol, followed by heating at 180°C. To reanalyze an unusual product (Xα, see Results section), the hydrolyzed products were also developed according to the method of Kolenová et al. (2006).

Structure of the hydrolyzed products with a molecular mass of more than 550 was analyzed by MALDI-TOF-MS according to the method of Reis et al. (2003). A sample for MALDI-TOF-MS analysis was prepared by mixing 1 μL of the products described above, 1 μL of 2,5-dihydroxybenzoic acid dissolved in a solvent mixture composed of 0.1% trifluoroacetic acid solution–acetonitrile (30:70, v/v), and 1 μL of 5-chloro-2-mercaptobenzothiazole dissolved in tetrahydrofuran–ethanol–water (1:1:1, v/v/v).

## Results

### Cloning of the xylanase genes of *A. niger* E-1 and phylogenetic analysis

We found eight putative xylanase genes on the *A. niger* CBS 513.88 genome and designated them *xynI*–*VIII* in ascending order of molecular masses of the deduced amino acid sequences (Table 1). To confirm the presence of these xylanase genes on the *A. niger* E-1 genome, PCR was performed using primer pairs designed on the basis of the putative *A. niger* CBS 513.88 xylanase gene sequences (Table 2). The seven putative xylanase genes *xynI*–*VII* were amplified and their nucleotide sequences showed 98.4%–100% identities with those of corresponding genes on the *A. niger* CBS 513.88 genome (data not shown). We found that *xynV*, showing the lowest identity (98.4%) in comparisons of corresponding nucleotide sequences, was found to possess substitutions of amino acid residues at three positions (A108→T, A110→T, and S161→T in the XynV sequence from *A. niger* CBS 513.88), and the Asn-38 residue in the XynII sequence of strain CBS 513.88 was also substituted with an Asp residue at the corresponding position in XynII of strain E-1 (99.7% identity in comparison of nucleotide sequence). The other xylanases had no substitutions. We could not amplify strain E-1 *xynVIII* by PCR using the pair of xynVIII2F and xynVIII2R which were designed from the internal sequence of strain CBS 513.88 *xynVIII*, in addition to the pair of xynVIII1F and xynVIII1R.

**Figure 1:**
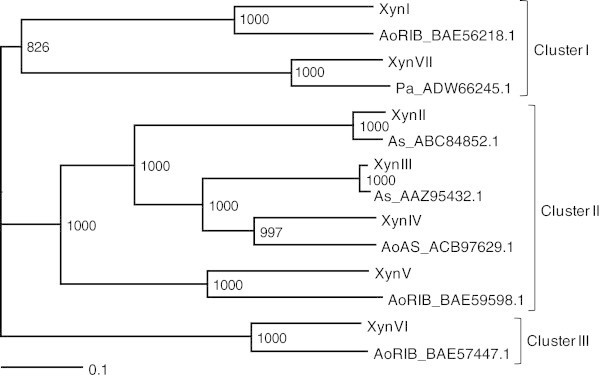
**Phylogenetic tree of xylanases from*A. niger*E-1 and the closest related sequences to strain E-1 xylanases.** The sequence names consist of the abbreviation of microorganisms and protein ID. Abbreviations: AoRIB, *Aspergillus oryzae* RIB40; AoAS, *Aspergillus oryzae* strain AS 3.4382; As, *Aspergillus sulphureus*; Pa, *Paecilomyces aerugineus*.

A phylogenetic tree based on the deduced amino acid sequences of *A. niger* E-1 xylanases was constructed using the Neighbor-Joining method (Figure 1). We found that the *A. niger* E-1 xylanases constitute independent three clusters. Cluster I consisted of XynI and XynVII although they branched deeply; XynII, XynIII, XynIV, and XynV, which belonged to GH family 11, were classified into another branch, cluster II; and only XynVI was localized in cluster III in the phylogenetic tree.

### Separation and identification of xylanases from strain E-1

**Figure 2:**
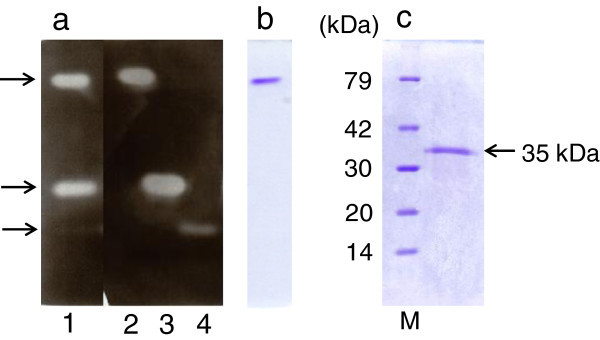
**PAGE of xylanase from*A. niger*E-1. (a)** Zymogram analysis of crude enzyme preparation (1.6 U, lane 1), fraction 1 (0.3 U, lane 2), fraction 2 (1.9 U, lane 3), and fraction 3 (1.8 U, lane 4). **(b)** Native-PAGE of fraction 1 (3.2 μg). **(c)** SDS-PAGE of fraction 1 (2.5 μg). Lane M, molecular mass standard proteins: lysozyme (14 kDa), trypsin inhibitor (20 kDa), carbonic anhydrase II (30 kDa), aldolase (42 kDa), and bovine serum albumin (79 kDa).

**Figure 3:**
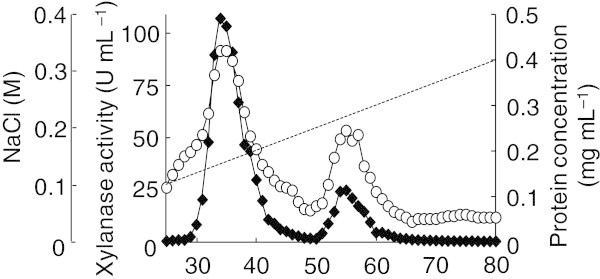
**Elution profile of xylanases by DE52 column chromatography.** Xylanase activity (*filled diamond*) and protein concentration (*open circle*) were assayed for each fraction.

*A. niger* E-1 produced three xylanases in the culture supernatant when the strain was cultured in 0.5% beechwood xylan medium supplemented with 50 mM sodium succinate (Figure 2a). In order to separate these xylanases, a crude enzyme preparation (4,500 U) was concentrated by lyophilization and applied to a DE52 column after dialysis. An eluate passing through the DE52 column showed xylanase activity and was stored as fraction 1. Two active peaks (fractions 2 and 3), which were eluted with 0.14 or 0.22 M sodium chloride, respectively, were observed when proteins were eluted from the column with a linear gradient of 0–0.5 M sodium chloride (Figure 3). The total activities of fractions 1, 2, and 3 were 670 U, 2,300 U, and 500 U, respectively, and their total amount was calculated as 77% of the crude enzyme preparation (4,500 U).

Zymogram analysis revealed that each fraction contained the only one active protein that was represented by a clear zone and corresponded to those in the culture supernatant (Figure 2a). Furthermore, fraction 1 showed only a single band by native- and SDS-PAGE (Figure 2b and 2c, respectively). These results indicate that the three extracellular xylanases produced by *A. niger* E-1 could be separated from each other, and the xylanase contained in fraction 1 was purified to homogeneity. By contrast, fractions 2 and 3 were found to contain some proteins by native- and SDS-PAGE (data not shown). The purification procedure for fraction 1 is summarized in Table 3. Fraction 1 was purified 1.4-fold with a specific activity of 99 U mg^–1^ and a yield of 15% of total activity in the culture supernatant.

**Table 3:** **Summary of XynVII purification**

Purification step	Total act. (U)	Total protein (mg)	Specific act. (U mg^–1^)	Yield (%)	Purification fold
Crude enzyme	4,500	65	69	100	1.0
Lyophilization	4,900	34	140	110	2.0
Anion-exchange (DE52)	670	6.8	99	15	1.4

Xylanases contained in fractions 1, 2, and 3 were identified by N-terminal amino acid sequencing or MALDI-TOF-MS analysis. N-terminal amino acid sequencing was not available for the identification of xylanase contained in fraction 1 because there were no significant signals of phenylthiohydantoin-amino acid derivatives released from an N-terminal amino acid residue. A trypsinized single band from fraction 1 was analyzed by MALDI-TOF-MS and resulting sequences were queried against the SwissProt database. As a result, 16 peptides were found to exactly correspond to the deduced amino acid sequence of XynVII. The molecular mass of the purified XynVII was 35 kDa by SDS-PAGE (Figure 2c). The isoelectric point of the enzyme was estimated to be 7.0. Furthermore, we characterized XynVII because information on the enzymatic properties of XynVII was limited.

When a protein with a molecular mass of 23 kDa in fraction 2 was subjected to N-terminal amino acid sequencing, a STPSSTGENNGFYYSFWTDG sequence was identified and found in the deduced amino acid sequence of *xynIII*, with an estimated molecular mass of 24 kDa. A protein with a molecular mass of 27 kDa in fraction 3 showed the N-terminal sequence of SAGINYVQNYNGNLGDFTYDESTG, which was contained in the deduced amino acid sequence of *xynII* with an estimated molecular mass of 23 kDa. Further N-terminal amino acid sequencing of other proteins electroblotted from fractions 2 and 3 revealed that they were not putative xylanases, but were other enzymes related to xylan degradation, for example, α-L-arabinofuranosidase and acetyl xylan esterase. These results strongly suggest that XynII and XynIII reflect xylanase activities obtained in fractions 2 and 3, respectively.

### The effects of pH and temperature on xylanase activity and stability

**Figure 4:**
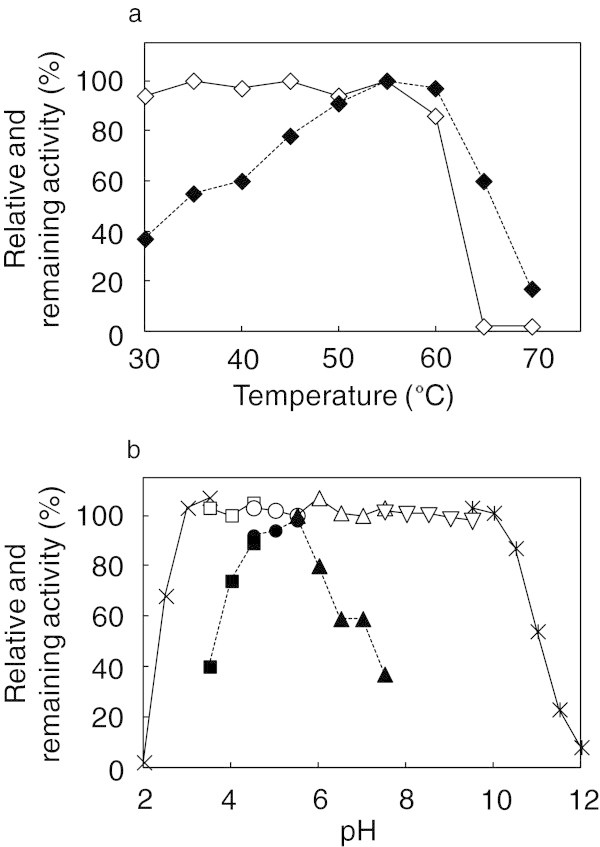
**Effects of temperature and pH on XynVII activity. (a)** Effects of temperature (*filled diamond*) and thermostability (*open diamond*). **(b)** Effects of pH (*filled symbols*, *dotted line*) and pH-stability (*open symbols*, *solid line*). The buffers used were 150 mM of glycine–HCl (*cross*), sodium acetate–HCl (*filled square*, *open square*), acetate (*filled circle*, *open circle*), sodium–potassium phosphate (*filled triangle*, *open triangle*), Tris–HCl (*inverted open triangle*), and glycine–NaOH (*asterisk*).

XynVII was the most active at 55°C, and its activity rapidly decreased above 60°C. The enzyme maintained more than 85% remaining activity after incubation at 30°C–60°C for 30 min (Figure 4a). The optimal pH for XynVII activity was 5.5, and this enzyme showed more than 70% relative activity in a mildly acidic pH range (pH 4–6) (Figure 4b). Furthermore, the enzyme was stable (approximately 100%) in the broad pH range of 3–10 and retained more than 50% of its activity after incubation at pH 2.5, 10.5, or 11 for 24 h (Figure 4b).

### Effects of metal ions and modifying reagents

The effects of various metal ions and modifying reagents on XynVII activity were determined. Hg^2+^ and *N*-bromosuccinimide (NBS) strongly inhibited xylanase activity (67% and 5% relative activity, respectively). By contrast, we found that the following metal ions and modifying reagents showed no remarkable effects (relative activity, 86–109%): Ba^2+^, Ca^2+^, Cd^2+^, Co^2+^, Cu^2+^, Fe^2+^, Mg^2+^, Mn^2+^, Ni^2+^, Pb^2+^, Zn^2+^, 4-chloromercuribenzoic acid, EDTA, and SDS.

### Substrate specificity and kinetic parameters

XynVII showed high specificity against beechwood xylan compared with oatspelt xylan (68% relative activity), whereas other hemicellulose-related substrates such as pectin, PNPAra, and PNPX were not suitable substrates for this enzyme (less than 2% relative activity). Moreover, the enzyme showed little activity against dextran, dextrin, or cellulolytic substrates such as CMC, crystalline cellulose, PNPC, or PNPG (less than 2% relative activity). The *K*_m_, *V*_max_, and *k*cat values of XynVII were 2.8 mg mL^–1^, 127 μmol min^–1^mg^–1^, and 76 s^–1^, respectively, when beechwood xylan was used as a substrate.

### Hydrolysis of beechwood xylan and xylooligosaccharides by purified XynVII

**Figure 5:**
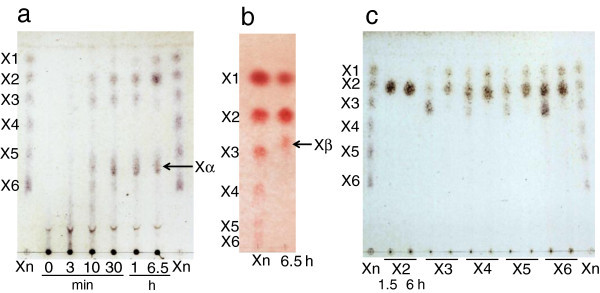
**TLC analysis of hydrolysates.** After hydrolysis of beechwood xylan **(a** and **b)** and xylooligosaccharides **(c)** with XynVII for various reaction times, hydrolysates were developed on a silica **(a** and **c)** and cellulose plate **(b)**. Abbreviations: X1, xylose; X2, xylobiose; X3, xylotriose; X4, xylotetraose; X5, xylopentaose; X6, xylohexaose; Xn, standards mixture of X1–X6. Unknown products are indicated by Xα and Xβ.

In order to analyze reaction products, beechwood xylan and various xylooligosaccharides (X2–X6) were hydrolyzed with purified XynVII, and the reaction products were analyzed by TLC using a silica gel plate and the solvent system of acetonitrile–ethyl acetate–2-propanol–water (17:5:11:10, v/v/v/v) (Figure 5a). For beechwood xylan, the enzyme initially released xylooligosaccharides with high molecular masses [degree of polymerization (DP) of >5], and the molecular masses became smaller (DP, 1–6) as the enzyme reaction progressed. Finally, the products converged on X1, X2, and Xα, which was localized between X5 and X6. When the final products were developed on a cellulose plate using the solvent system of ethyl acetate–acetic acid–water (3:2:2, v/v/v), Xβ appeared at a position between X2 and X3, instead of Xα, in addition to X1 and X2 (Figure 5b). The position of Xβ was similar to that of X3 substituted with a 4-*o*-methylglucuronic acid residue (X3MeGlcA) that was reported by Kolenová et al. (2006). In hydrolysis tests of xylooligosaccharides, the enzyme hydrolyzed X3–X6 and produced X1 and X2 as the final hydrolysates after a 6-h incubation, but no reaction products were observed when X2 was used as a substrate (Figure 5c).

To identify the final product (Xβ) appearing at an unusual position in TLC analysis, MALDI-TOF-MS analysis was performed. The MALDI mass spectra obtained from the product Xβ showed more abundant ions at *m/z* 627 and 649. These ions could be attributed to xylotriose substituted with a 4-*o*-methylglucuronic acid residue (X3MeGlcA), corresponding to a 23 and 22 Da increase that could be attributed to a single- or double-sodiated ion (*m/z*). The ions observed at *m/z* 649 resulted from the substitution of one hydrogen atom by a second sodium atom (Reis et al. 2003). In conclusion, we identified the final reaction products Xα and Xβ as X3MeGlcA based on the results of TLC and MALDI-TOF-MS analyses.

## Discussion

Thus far many researchers have reported enzymatic properties, gene cloning, and heterologous expression of individual xylanases from *A. niger*. However, less attention has been paid to the relationship between xylanase genes carried on *A. niger* genome and xylanases produced. Therefore, we examined the xylanase genes on *A. niger* E-1 genome that contribute to xylan degradation though the biosynthesis of functionally active xylanases.

The *A. niger* E-1 genome encoded seven putative xylanase genes *xynI*–*VII*, which are closely similar to those of xylanase genes from *A. niger* CBS 513.88. On the other hand, strain E-1 produced three xylanases, XynII, XynIII, and XynVII, when this strain was cultured in 0.5% xylan medium supplemented with 50 mM sodium succinate (Figure 2a and Figure 3). There are no reports, to our knowledge, on the expression of *xynIV* and *xynV* belonging to cluster II in a phylogenetic tree (Figure 1) or *xynI* and *xynVI* in other *A. niger* strains. These results suggest that these xylanases may play physiologically distinct roles from XynII, XynIII, and XynVII to adapt to quite different environments and assimilate xylan.

XynIII activity represented 51% of total activity in the culture supernatant; XynII and XynVII activities were 15% and 11%, respectively, of the total activity, suggesting that XynIII plays a primary role in the degradation of xylan backbones in this culture condition. This speculation is also supported by the fact that many XynIII and closely related enzymes have been reported from *A. niger* strains (Krisana et al. 2005; Fu et al. 2012). In addition to XynIII, some enzymes highly homologous to XynII have also been reported (Hmida-Sayari et al. 2012; Yang et al. 2010), but information on XynVII, which belongs to GH family 10 in *A. niger*, was limited. Therefore, we characterized XynVII in further experiments.

Aspergilli endoxylanases show the maximal activities at a range of 42°C–60°C, and a pH range of 4.0–7.0 (Teixeira et al. 2010). The highest activity of purified XynVII was also observed within these ranges. However, XynVII maintained more than 85% activity after 30 min of incubation at 60°C (Figure 4a), and was more stable than other xylanases from *A. niger* strains US368 (Hmida-Sayari et al. 2012) and A-25 (Chen et al. 2006), which retained 50% and 10% of activities, respectively. Moreover, the pH stability profile showed that XynVII is highly stable over a wide pH range from 3.0 to 10.0 (Figure 4b), compared with other reported xylanases from *A. niger* strains (Fu et al. 2012; Hmida-Sayari et al. 2012; Krisana et al. 2005; Yang et al. 2010). In addition, *K*_m_ and *V*_max_ values of XynVII (2.8 mg mL^–1^ and 127 μmol min^–1^mg^–1^, respectively) were similar to those of GH family 10 xylanases from *Penicillium pinophilum* strain C1 [4.3 mg mL^–1^ and 195.4 μmol min^–1^mg^–1^, respectively (Cai et al. 2011)] and of GH family 11 xylanase from *A. niger* strain US368 [1.03 mg mL^–1^ and 811 μmol min^–1^mg^–1^, respectively (Hmida-Sayari et al. 2012)], indicating that XynVII from strain E-1 possesses catalytic properties that are sufficient to contribute to xylan degradation although the total activity of XynVII was smaller than those of other xylanases in the culture supernatant.

As reported for various xylanases from *A. niger* (Chen et al. 2006; Hmida-Sayari et al. 2012; Yang et al. 2010) and GH family 10 xylanase from *Flavobacterium johnsoniae* (Chen et al. 2013), XynVII activity was strongly inhibited by Hg^2+^, which potentially caused inhibition by its interaction with an aromatic ring present in a Trp residue. This finding was consistent with the result that XynVII was strongly inhibited by NBS, which modifies a Trp residue. It is interesting that XynVII was stable in the presence of other metal ions and modifying reagents, particularly Cu^2+^ and Mn^2+^, which are well-known inhibitors of xylanases (Chen et al. 2006; Hmida-Sayari et al. 2012).

The N-terminal amino acid sequence determination of purified XynVII was unsuccessful by a gas-phase protein sequencing. Liu et al. (2012) have studied a signal peptide cleavage site in the N-terminal region of *A. niger* XynB, which is identical to that of XynVII, and proposed that the cleavage of a peptide bond occurs between Arg-25 and Gln-26, releasing the signal peptide. It is likely that the N-terminal Gln residue (Gln-26) of mature E-1 XynVII is changed to pyroglutamate by cyclization after hydrolysis of the peptide bond, yielding a blocked N-terminus (Ito et al. 1992).

GH family 10 and GH family 11 xylanases differ in produced xylooligosaccharides. When a glucuronoxylan, such as beechwood xylan, is digested with GH family 11 xylanases, xylotetraose substituted with a 4-*o*-methylglucuronic acid residue (X4MeGlcA) accumulates as the final reaction product (Biely et al. 1997; Kolenová et al. 2006). X4MeGlcA generally show resistance against hydrolysis with xylosidase, when substitution by glucuronic acid occurs at X1 of the non-reducing end or the second X1 next to the non-reducing end (Tenkanen et al. 1996; Rasmussen et al. 2012). Although glucuronic acid is released from short xylooligosaccharides by the actions of α-glucuronidase, X4MeGlcA is too large to be hydrolyzed with the enzyme (Kolenová et al. 2006). As a result, it is assumed that X4MeGlcA remains and complete glucuronoxylan degradation is unsuccessful when glucuronoxylan is digested with GH family 11 xylanases. On the other hand, X3MeGlcA, which is produced by GH family 10 xylanase reaction, is hydrolyzed with α-glucuronidase, and the resulting X3 is acceptable for hydrolysis of xylosidase. Thus, the GH family 10 xylanase XynVII appears to contribute to efficient glucuronoxylan assimilation by strain E-1. Zheng et al. (2013) recently cloned a cDNA encoding GH family 10 xylanase from *A. niger* and characterized the purified recombinant enzyme. Characteristics of the recombinant enzyme were similar to those of E-1 XynVII. In addition to these characteristics, we identified the reaction products of E-1 enzyme and suggested the physiological significance of XynVII in glucuronoxylan assimilation based on the reaction products as described above.

In this study, we found only one gene encoding GH family 10 xylanase and four genes encoding GH family 11 xylanases from *A. niger* E-1. Wakiyama et al. (2008) showed that GH family 10 xylanases from *Penicillium* spp. and *Aspergillus* spp. could be classified into two groups by phylogenetic analysis: one is a xylanase group from *Penicillium* spp. and black aspergilli such as *A. kawachii* and *A. niger*, and another is from other aspergilli, such as *A. fumigatus*, *A. nidulans*, and *A. oryzae*. In the aspergilli described above, it has been reported that *A. fumigatus*, *A. nidulans*, and *A. oryzae*, of which the genomes have been sequenced, possess more than two GH family 10 genes on their genome. By contrast, the genome of the black aspergillus *A. niger* carried only one GH family 10 xylanase gene in both strains E-1 and CBS 513.88. Genome information about other black aspergilli is not available for database search, but numbers of GH family 10 xylanase genes on each aspergillus genome may be related to the diversity of GH family 10 xylanases in the evolution processes of aspergilli genomes.

*A. niger* E-1 possesses seven putative xylanase genes (*xynI*–*VII*), but only three xylanases (XynII, XynIII, and XynVII) were produced, strongly suggesting that potential regulatory mechanisms control the expression of the other four xylanase genes, *xynI*, *xynIV*, *xynV*, and *xynVI*, for which no translated products were detected. Further research is needed to comprehensively understand xylan degradation by *A. niger* E-1.
